# The impact of a computerized physician order entry system implementation on 20 different criteria of medication documentation—a before-and-after study

**DOI:** 10.1186/s12911-021-01607-6

**Published:** 2021-10-11

**Authors:** Viktoria Jungreithmayr, Andreas D. Meid, Implementation Team, Janina Bittmann, Markus Fabian, Ulrike Klein, Silvia Kugler, Martin Löpprich, Oliver Reinhard, Lucienne Scholz, Birgit Zeeh, Wolfgang Bitz, Till Bugaj, Lars Kihm, Stefan Kopf, Anja Liemann, Petra Wagenlechner, Johanna Zemva, Claudia Benkert, Christian Merle, Sergej Roman, Stefan Welte, Walter E. Haefeli, Hanna M. Seidling

**Affiliations:** 1 Department of Clinical Pharmacology and Pharmacoepidemiology, Heidelberg University Hospital, Im Neuenheimer Feld 410, 69120 Heidelberg, Germany; 2 Cooperation Unit Clinical Pharmacy, Heidelberg University Hospital, Im Neuenheimer Feld 410, 69120 Heidelberg, Germany; 3 Heidelberg University Hospital, Im Neuenheimer Feld 672, 69120 Heidelberg, Germany

**Keywords:** Computerized physician order entry system, Evaluation results, Medication prescription, Documentation errors

## Abstract

**Background:**

The medication process is complex and error-prone. To avoid medication errors, a medication order should fulfil certain criteria, such as good readability and comprehensiveness. In this context, a computerized physician order entry (CPOE) system can be helpful. This study aims to investigate the distinct effects on the quality of prescription documentation of a CPOE system implemented on general wards in a large tertiary care hospital.

**Methods:**

In a retrospective analysis, the prescriptions of two groups of 160 patients each were evaluated, with data collected before and after the introduction of a CPOE system. According to nationally available recommendations on prescription documentation, it was assessed whether each prescription fulfilled the established 20 criteria for a safe, complete, and actionable prescription. The resulting fulfilment scores (prescription-*Fscores*) were compared between the pre-implementation and the post-implementation group and a multivariable analysis was performed to identify the effects of further covariates, i.e., the prescription category, the ward, and the number of concurrently prescribed drugs. Additionally, the fulfilment of the 20 criteria was assessed at an individual criterion-level (denoted criteria-*Fscores*).

**Results:**

The overall mean prescription-*Fscore* increased from 57.4% ± 12.0% (n = 1850 prescriptions) before to 89.8% ± 7.2% (n = 1592 prescriptions) after the implementation (*p* < 0.001). At the level of individual criteria, criteria-*Fscores* significantly improved in most criteria (n = 14), with 6 criteria reaching a total score of 100% after CPOE implementation. Four criteria showed no statistically significant difference and in two criteria, criteria-*Fscores* deteriorated significantly. A multivariable analysis confirmed the large impact of the CPOE implementation on prescription-*Fscores* which was consistent when adjusting for the confounding potential of further covariates.

**Conclusions:**

While the quality of prescription documentation generally increases with implementation of a CPOE system, certain criteria are difficult to fulfil even with the help of a CPOE system. This highlights the need to accompany a CPOE implementation with a thorough evaluation that can provide important information on possible improvements of the software, training needs of prescribers, or the necessity of modifying the underlying clinical processes.

**Supplementary Information:**

The online version contains supplementary material available at 10.1186/s12911-021-01607-6.

## Background

The occurrence of medication errors in hospitals is known to be a common and potentially serious threat to patient safety [1, 2]. While medication errors can occur at all stages of the medication process, prescribing errors are particularly common [3] and often caused by incorrect documentation of intended medication orders [4]. Manual, paper-based prescribing is still a significant error source as many errors are due to illegible handwriting or omitted data, such as missing dosage information, forgotten units of measure, or an incomplete route of administration. These errors are either due to prescribing oversights or to a lack of information or knowledge [5]. There are a number of guidelines defining the minimum standards that a drug prescription should meet, e.g., regarding comprehensive prescription documentation [4, 6–8]. These guideline standards can often be met through the implementation of a computerized physician order entry (CPOE) system. Therefore, these systems are frequently proposed as an important element to increase medication safety [5, 9–15].

Until now, the benefits of CPOE systems have often been assessed by evaluating medication errors. However, the reduction of medication error rates commonly stagnates at 50%, suggesting that CPOE systems can eliminate some but not all errors. In several retrospective analyses the implementation of CPOE systems most frequently decreased the medication error rate by eliminating illegible orders [16]. Furthermore, CPOE systems have a significant potential to reduce ambiguous prescriptions and omission of data as common sources of error [12]. On the other hand, CPOE systems can introduce new errors, especially due to system-user interface deficiencies, misleading computer screen displays, incorrect workflows, or due to a poor use of the system [17, 18]. Medication errors fostered by CPOE systems can result from incorrect medication selection from drop-down menus, incorrect data placement [19, 20], and failures to set default CPOE settings [19]. To sum up, after extensive knowledge on the quantity and type of medication errors has already been gathered, we wanted to take an even deeper look into different quality criteria of medication documentation. This gives us more insights into potential error-sources and omissions in medication documentation and allows us to identify appropriate preventive measures. These insights are highly valuable when further improving the safety of CPOE systems and their usage.

The aim of this study was to comprehensively assess the impact of a CPOE system on medication documentation, to determine which documentation criteria can be improved by the implementation of a CPOE and to examine how this change is influenced by concomitant factors.

## Methods

### Setting

This study was conducted at Heidelberg University Hospital, a large tertiary care hospital where an electronic health record (EHR; Cerner® i.s.h.med (SAP release EhP8, Support Package 016-024)) was newly equipped with a CPOE system including an integrated clinical decision support system (CDSS) in December 2018. The study was approved by the responsible Ethics Committee of the Medical Faculty of Heidelberg (S-453/2019) and by the staff council of Heidelberg University Hospital. Informed consent could be dispensed with since analyses focused on prescription data (thereby using routinely documented information) and did not assess any outcomes on patient or prescriber level.

Seven out of 71 general wards are currently equipped with the CPOE system and were included in this evaluation. The seven evaluated pilot wards have a maximum capacity of 184 beds, divided among radio-oncology wards (60 beds), surgical-orthopaedic wards (specialised in endoprosthetics and spine surgery, 52 beds) and internal medicine wards (specialised in endocrinology, cardiology, and psychosomatic medicine; 72 beds). The workflow of medication documentation differed between the wards before the CPOE implementation, with both nurses and physicians being involved. Prescriptions on paper charts were either documented by physicians themselves or by nurses, based on instructions by a physician. Changes to the current prescriptions were likewise either documented by physicians themselves or by nurses, based on instructions by a physician. Discharge medication was documented by the physicians in an electronic system that automatically transferred medication prescriptions to the discharge letters. On the contrary, medication documentation is solely a physicians’ task and is performed in the same way on every ward after the CPOE implementation. Physicians are responsible for documenting all medication prescriptions, changes to them and the discharge medication in the CPOE system.

### Study population

We conducted a retrospective data analysis considering in-house drug prescriptions of 160 patients before (pre-implementation cohort) and of another set of 160 patients after CPOE implementation (post-implementation cohort). On each ward, prescriptions from 20 patients per time point were included in the study. An exception was made for one exceptionally large ward with twice the number of beds where prescriptions of 40 patients were analysed. One to three months before the CPOE implementation, successive patients with at least one drug prescription and an available scan of their paper chart in the electronic archive were included as baseline assessment. One to three and a half months after implementation, successive patients with at least one drug prescription and an available electronic chart were included. To include the fix number of patients, screening periods differed between wards with shorter screening periods on wards with a high patient throughput and longer screening periods on wards where patients stayed longer. To ensure comparability of the cohorts, the post-implementation cohort included only patients whose number of total prescriptions, standard peroral prescriptions, prescriptions with a risky administration route, prescriptions as needed, and other prescriptions were within one standard deviation of the average calculated from the pre-implementation cohort.

### Data collection

For all included patients, the prescription data documented on their second inpatient treatment day were extracted. Demographic data collected included age, sex, weight, renal function (serum creatinine and estimated glomerular filtration rate (calculated by means of CKD-EPI equation)), and the ward to which the patient was admitted. To compile the pre-implementation dataset, the electronic archive was screened and prescription and demographic data (age, sex, weight, ward) were manually extracted from scanned paper charts. Additionally, information on patients’ renal function was extracted from the electronic laboratory system. Post-implementation, logged prescription data from the CPOE system were retrieved along with manually extracted demographic data from the electronic chart (age, sex, weight, ward) and the electronic laboratory system (renal function). Total prescriptions per patient and their distribution across different prescription categories (standard peroral prescriptions, prescriptions with a risky administration route, prescriptions as needed, and other prescriptions) were counted. Any medication prescribed “as needed” was counted in the prescriptions-as-needed group, regardless of the administration route. All prescriptions with a regular administration scheme were classified into one of the other groups, based on the route of administration. This means that every regular prescription with a peroral administration route was classified as “standard peroral prescription”, every regular prescription with a risky administration route as defined in Table 1 was classified as “prescription with a risky administration route” and every regular prescription with another than peroral or risky administration route (e.g., transdermal, ocular, nasal) was classified as “other prescription”.

**Table 1:** Administration routes classified as “risky administration route”

Endotracheal	Endo-cervical	Epidural
Epilesional	Extra-amniotic	Gastrointestinal
Gingival	All administration types with the prefix intra-	Laryngopharyngeal
Ossal	Para-cervical	Periarticular
Peribulbar	Perineural	Periosteal
Retrobulbar	Sub-tenon	Subconjunctival
Subcutaneous	Sublesional	Submucosal
Urethral	Via probe	

### Data appraisal

The prescriptions were assessed according to the recommendation “Good prescribing practice in drug therapy” published by the Aktionsbündnis Patientensicherheit e.V. (english: alliance for patient safety, APS) [21], a German interprofessional non-profit organization advocating measures to enhance patient safety. The recommendation on good prescribing practice is based on international guidelines and consists of 20 explicit criteria that every prescription should fulfil to be safe and actionable. The first five criteria ask for the presence of relevant patient data (allergies and intolerances, age in years, weight, renal function and drug history) that is needed to evaluate the adequacy of a prescription. Criteria #6 to #15 ask for formal requirements of a complete prescription (e.g., validity, readability, provision of comprehensive information on the drug and the dosage). Criteria #16 to #20 pose clinical questions and ask for information to enable safe administration of a drug. They also determine whether the prescription poses any risks for the patient and whether the prescription is actionable and unambiguous for the person supposed to administer the drug. Each criterion listed in the recommendation was either rated as met, not met, or not applicable. Based on this rating, two different scores that indicate the fulfilment of criteria have been calculated. One at the prescription-level (prescription-*Fscore*) and another one at the criteria-level (criteria-*Fscore*). When analysing the prescriptions accordingly, a score that indicates the percentage of fulfilled criteria per prescription (fulfilment score, denoted prescription-*Fscore* henceforth) was calculated as the following: $\frac{{{\text{number}}\,{\text{of}}\,{\text{met}}\,{\text{criteria}}}}{{{\text{number }}\,{\text{of}}\,{\text{applicable}}\,{\text{criteria}}}}$. This prescription-*Fscore* was used for the comparison between the different time points of the analysis (before and after the CPOE implementation) for all prescriptions and separately for every individual prescription category. Additionally, all prescription-*Fscores* were included in a multivariable analysis (for more details, see statistical analysis section). Moreover, an assessment on criteria-level was performed to gain insight on whether the CPOE-implementation influenced the fulfilment of any of the 20 criteria in a positive or negative way. This score (denoted criteria-*Fscore* henceforth) was calculated for every criterion at each time point, respectively as: $\frac{{{\text{number}}\,{\text{of}}\,{\text{prescriptions }}\,{\text{that}}\,{\text{met}}\,{\text{this}}\,{\text{criterion}}}}{{{\text{number}}\,{\text{of}}\,{\text{prescriptions}}\,{\text{for}}\,{\text{which}}\,{\text{this}}\,{\text{criterion}}\,{\text{was }}\,{\text{applicable}}}}{.}$ The analysis was performed by the principal investigator (VJ), and 10% of the data were double-checked by sub-investigators. When the evaluation of the double-checked prescriptions revealed discrepancies, these were discussed. If this resulted in changes to the general evaluation scheme, all relevant evaluations were changed accordingly. In case of any unforeseen deviation from the evaluation scheme, the double check was extended to the entire data set. The detailed assessment scheme can be found under Supplementary Information (Additional file 1). All prescriptions were reviewed for drug-drug interactions, allergies, duplicate prescriptions, potentially inappropriate medication for the elderly, dose adjustment for renal function, and maximum approved dose (AiD*Klinik*®, Dosing GmbH, Heidelberg, Germany, data version 01.12.2019).

### Statistical analysis

Standard statistical methods were applied to describe population characteristics. Comparisons of prescription-*Fscores* were tested using Mann–Whitney U-Test and frequency distributions of the fulfilment of individual criteria (criteria-*Fscores*) with Chi-squared test.

All prescription-*Fscores* are included as outcome variables in a multivariable analysis with each prescription as the observation unit. The prescription-*Fscore* is a proportion and thus bounded at both ends of the scale and potentially skewed. The beta distribution not only fits such data distributions better than the normal distribution, beta regression models also account for the boundedness of the outcome variable [22]. To overcome the potential limitation of values at the boundaries, we chose the common continuous transformation [23, 24] to transform the prescription-*Fscore* in our sample of totally *N* = 3442 observations:

$$\left[ {prescriptionFscore*{ } \left( {N - 1} \right) + 0.5} \right]{/}N$$

With regard to the particular prescription-*Fscores*, the observation units (assessed prescriptions) are clustered within the sampling units (patients) so that assessments within the same patient are typically correlated (and thus violate the basic assumption of conditionally independent observations). In particular, we observe the fulfilment score $prescription{Fscore}_{ij}$ for $j$ medications nested within $i$ patients. Extensions of beta regression models to beta-distributed generalized linear mixed models (GLMM) allow adding $b_{i}$ as a patient-specific random effect to account for intra-patient correlations [24, 25]:

$$\log \left( {\frac{{prescriptionFscore_{ij} }}{{1 - prescriptionFscore_{ij} }}} \right) = x_{ij}^{T} \beta + z_{ij}^{T} b_{i} \,\,\,\,with\, b_{i} \sim N\left( {0,G} \right)$$

$x_{ij}^{T}$ and $z_{ij}^{T}$ denote vectors of data (covariates) for the estimation of fixed parameter effects $\beta$ and within-patient correlations $b_{i}$ (with their covariance matrix G). Data variables in our random-intercept model were *time point* (post-implementation versus pre-implementation), *prescription categories* (reference: standard peroral prescriptions), the discrete *number of comedications* and the effect-coded *ward indicator* (weighted for the relative number of medications from the respective ward) [26].

It follows that parameter estimates in the beta regression model can be expressed and interpreted in terms of odds ratios (OR); we thus calculated the odds ratios for improving the ratio between the prescription-*Fscore* and the difference to the perfect scoring (1 − prescription-*Fscore*). Random-effects were estimated as standard deviations to explain the source of correlation [27]. Acknowledging that estimated effects are adjusted for individual differences thus referring to within-individual change, we additionally visualized the effect by predicting the covariate-adjusted prescription-*Fscore* for each observation from the data set.

Statistical analyses were conducted using IBM® SPSS® Statistics (Version 25) and the R software environment in version 4.0.2 (R Foundation for Statistical Computing, Vienna, Austria) with the key packages *betareg* (version 3.1-3) and *glmmTMB* (version 1.0.2.1).

## Results

### Patient characteristics

In total, 3442 prescriptions from 320 patients were evaluated (Table 2). The pre-implementation and post-implementation cohorts did not differ with regard to age, sex, number of standard peroral prescriptions, and number of prescriptions with a risky administration route. However, more prescriptions were collected in the pre-cohort, mainly due to more “as needed” prescriptions and more “other prescriptions”.

**Table 2:** General characteristics and demographics of the pre-implementation and post-implementation cohort

		Pre-implementation cohort (n = 160)	Post-implementation cohort (n = 160)	*P* value
Age	Mean value (SD)	58.2 (± 22.1)	59.8 (± 17.7)	0.854
Sex	Female	51.9%	49.4%	0.655
Average number of prescriptions per patient	Mean value (SD)	11.6 (± 5.6)	10.0 (± 3.3)	n.a.
All prescriptions	n (%)	1850 (100%)	1592 (100%)	0.009
Standard peroral prescriptions	n (%)	1051 (56.8%)	990 (62.2%)	0.673
Prescriptions with a risky administration route	n (%)	265 (14.3%)	200 (12.6%)	0.117
Prescriptions as needed	n (%)	452 (24.4%)	362 (22.7%)	0.012
Other prescription	n (%)	82 (4.4%)	40 (2.5%)	0.002

n, number; SD, standard deviation

### Analysis of prescription-fulfilment scores

A prescription-*Fscore* was calculated for all 3442 prescriptions. The average number of criteria that were not applicable was 3.4 criteria (± 0.8) per prescription. The quality assurance measures did not reveal any unforeseen discrepancies.

**Fig. 1:**
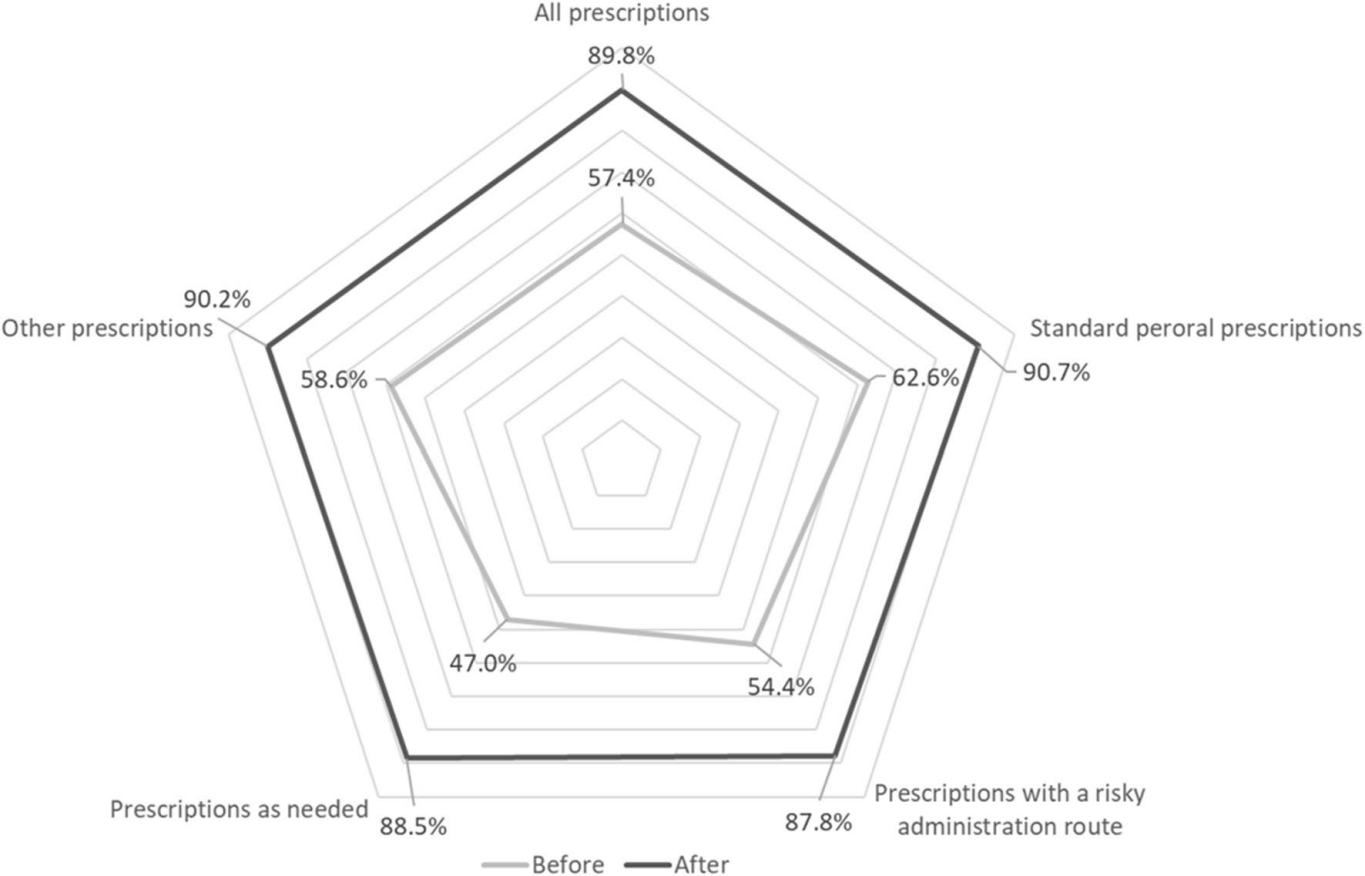
Comparison of prescription-*Fscores* in the pre-implementation cohort and post-implementation cohort for individual prescription categories. After: post-implementation cohort; before: pre-implementation cohort; CPOE: computerized physician order entry; prescription-Fscore: fulfilment score per prescription

The prescription-*Fscores* for all prescriptions increased significantly (*p* < 0.001) from 57.4% ± 12.0% (n = 1850 prescriptions) before to 89.8% ± 7.2% (n = 1592 prescriptions) after CPOE implementation. After CPOE implementation, a significant (*p* < 0.001) increase in prescription-*Fscores* was observed in each individual prescription category (Table 3, Fig. 1). A significant (*p* < 0.001) increase in prescription-*Fscores* with a large effect size (r > 0.5) could be seen on every ward after the CPOE implementation.

**Table 3:** Prescription-*Fscores* of all prescriptions and of individual prescription categories

	Paper-based		CPOE		*P* value
	Mean value (SD)	n	Mean value (SD)	n	
All prescriptions	57.4% (± 12.0%)	1850	89.8% (± 7.2%)	1592	< 0.001
Standard peroral prescriptions	62.6% (± 10.1%)	1051	90.7% (± 6.6%)	990	< 0.001
Prescriptions with a risky administration route	54.4% (± 10.4%)	265	87.8% (± 7.8%)	200	< 0.001
Prescriptions as needed	47.0% (± 9.6%)	452	88.5% (± 8.0%)	362	< 0.001
Other prescriptions	58.6% (± 11.3%)	82	90.2% (± 6.1%)	40	< 0.001

CPOE, computerized physician order entry; prescription-*Fscore*, fulfilment score per prescription; n, number; SD, standard deviation

### Multivariable analysis of prescription-Fscores

**Fig. 2:**
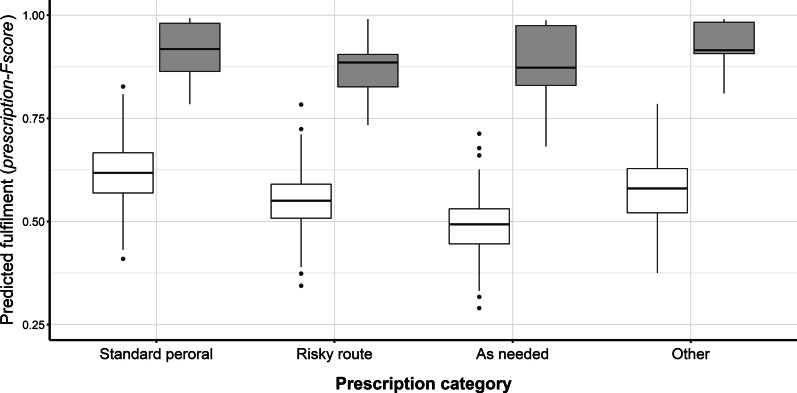
Boxplot of model-predicted prescription-*Fscores* in actual medications stratified for the categorized prescription categories. Pre-implementation group: open boxes; post-implementation group: grey boxes; prescription-*Fscore:* fulfilment score per prescription

Multivariable adjustment for potential confounders confirmed the large impact of the intervention on the prescription-*Fscores* as was already visible in the descriptive analyses (Table 4). Adjusted for the influence of the prescription category, ward, and number of concurrently prescribed drugs, the drugs prescribed with the CPOE system were over ten times (OR = 10.11 [95% CI 8.49–12.05]) more likely to achieve a higher prescription-*Fscore* when compared to paper-based prescriptions. Administration forms other than the standard peroral administration route were associated with lower prescription-*Fscores* (“risky route”, OR = 0.76 [95% CI 0.73–0.79]; “as needed”, OR = 0.59 [95% CI 0.57–0.61]; “other”, OR = 0.87 [95% CI 0.80–0.93]). Net absolute interventional effects expressed as differences in predicted group means were 29.6% (standard peroral prescriptions), 33.5% (prescriptions with a risky administration route), 39.3% (prescriptions as needed), and 35.4% (other prescriptions), respectively (Fig. 2). We also noted that the prescription-*Fscore* is a ward-dependent variable. The prescription-*Fscores* were significantly lower than the global (weighted) average of all wards at one ward (ward 3, OR = 0.53 [95% CI 0.43–0.67]), whereas three other wards (ward 5, OR = 1.29 [95% CI 1.03–1.63]; ward 6, OR = 1.44 [95% CI 1.11–1.86]; and ward 7, OR = 1.21 [95% CI 1.05–1.40]) had significantly higher prescription-*Fscores* than the global (weighted) average of all wards.

**Table 4:** Multivariable analysis of prescription-*Fscores* (outcome variables) estimated from a beta-distributed generalized linear mixed models (GLMM)

	Odds ratio	95% CI	*P* value
Baseline effect (intercept)	1.71	[1.31; 2.23]	< 0.001
Time point: post-implementation versus pre-implementation	10.11	[8.49; 12.05]	< 0.001
Prescription category (reference: standard peroral administration)			
Risky route	0.76	[0.73; 0.79]	< 0.001
As needed	0.59	[0.57; 0.61]	< 0.001
Other	0.87	[0.80; 0.93]	< 0.001
Number of comedications (n)	0.99	[0.97; 1.02]	0.622
Ward indicator^a^			
Ward 1	0.80	[0.64; 1.01]	0.057
Ward 2	1.04	[0.83; 1.31]	0.707
Ward 3	0.53	[0.43; 0.67]	< 0.001
Ward 4	0.82	[0.64; 1.05]	0.115
Ward 5	1.29	[1.03; 1.63]	0.028
Ward 6	1.44	[1.11; 1.86]	0.005
Ward 7	1.21	[1.05; 1.40]	0.009

CI, confidence interval; prescription-*Fscore*, fulfilment score per prescription

Within-patient correlation (random-effects standard deviation): 0.762 [95% CI 0.699–0.831]; Prescription-*Fscores* estimated as odds ratios with 95% confidence intervals (CI)

^a^Categories were included by weighted effect-coding[26]

### Analysis of individual criteria

When analysing the individual criteria, four criteria (#4, #15, #17, and #18) were unchanged after CPOE implementation, two criteria (#1 and #9) deteriorated in the criteria-*Fscores*, fourteen criteria (#2, #3, #5, #6, #7, #8, #10, #11, #12, #13, #14, #16, #19, and #20) increased, whereof six (#2, #6, #7, #8, #14, and #16) reached the maximum score of 100% (Table 5).

**Table 5:** Analysis of the criteria-*Fscores*

Criterion	Recommended criteria	Paper-based criteria-*Fscore* (absolute figures)	CPOE criteria-*Fscore* (absolute figures)	*P* value	Change pattern
Number	**The prescription includes relevant patient data:**				
1	Allergies and intolerances	81.9% (1515/1850)	54.0% (859/1592)	< 0.001	
2	Age in years	18.2% (337/1850)	100.0% (1592/1592)	< 0.001	
3	Weight in kilograms	55.2% (1021/1850)	87.0% (1385/1592)	< 0.001	
4	Organ functions (eGFR and creatinine)	88.9% (1645/1850)	88.0% (1401/1592)	0.401	
5	Drug history	43.1% (750/1742)	61.9% (921/1489)	< 0.001	
6	The prescription is valid	57.5% (1063/1850)	100.0% (1592/1592)	< 0.001	
7	The prescription was created with the aid of a computer	0.0% (0/1850)	100.0% (1592/1592)	< 0.001	
8	The prescription is easy to read	89.8% (1662/1850)	100.0% (1592/1592)	< 0.001	
9	The prescription does not contain abbreviations for active substances	78.8% (646/820)	73.4% (568/774)	0.012	
	**The prescription contains minimum information on the drug:**				
10	Full trade name or every active substance	96.2% (1780/1850)	99.7% (1588/1592)	< 0.001	
11	Dose strength and unit	57.1% (1053/1843)	80.0% (1269/1586)	< 0.001	
12	Dosage form and type of release	12.6% (234/1850)	96.4% (1535/1592)	< 0.001	
	**The prescription contains minimum dosage information:**				
13	Single dose and unit	14.9% (273/1833)	94.4% (1503/1592)	< 0.001	
14	Dosage interval	74.4% (1364/1833)	100.0% (1592/1592)	< 0.001	
15	The prescription contains the single dose as a whole number	96.9% (1363/1407)	97.7% (1470/1505)	0.183	
16	The prescription states invasive, risky administration types clearly and without abbreviations	8.4% (29/345)	100.0% (243/243)	< 0.001	
17	The prescription clearly indicates the body part and site for administration	27.3% (3/11)	60.0% (3/5)	0.299	
18	The prescription contains no identifiable risks for the patient	93.0% (1720/1850)	94.1% (1498/1592)	0.183	
19	The prescription contains the reason for administration	1.3% (6/452)	79.8% (289/362)	< 0.001	
20	The prescription is complete and unambiguous	61.9% (1145/1850)	91.4% (1455/1592)	< 0.001	

CPOE, computerized physician order entry; criteria-*Fscore*, fulfilment score per criterion;  , change pattern with different starting points, end points, and gradient direction;  , change pattern with different starting and end points but same gradient direction;  , change pattern with different starting points but same end points and gradient direction;  , change pattern with same starting points, end points, and gradient direction

### Change patterns of individual criteria

**Fig. 3:**
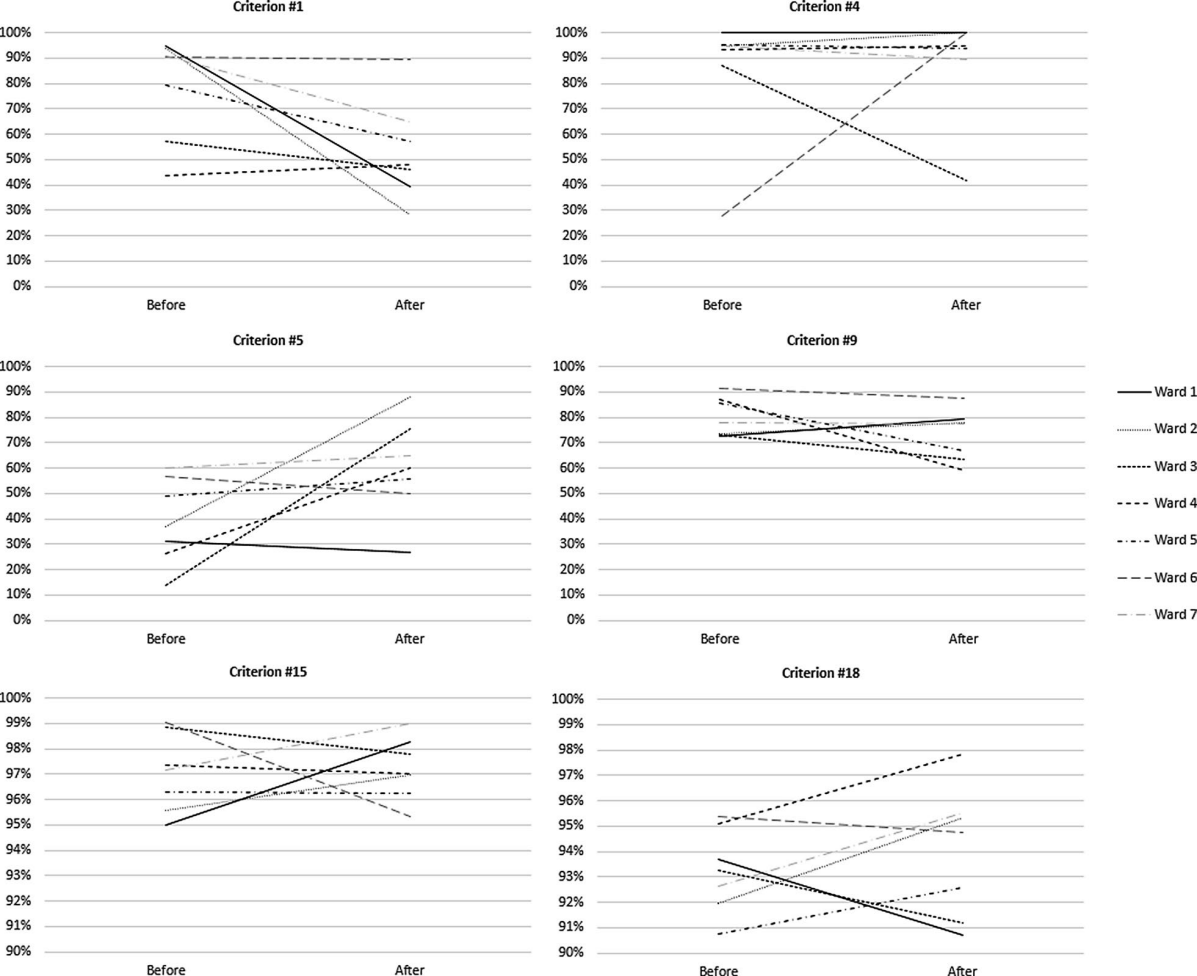
Individual criteria with different starting points, end points, and gradient direction. Criterion #17 is not reported here because this criterion was not applicable to every ward

Taking a deeper look into the criteria-*Fscores* of individual criteria, distinct differences in the change patterns between different wards can be found. Depending on the criterion, criteria-*Fscores* of different wards could show differences in starting points, end points, and gradient direction; different starting and end points but same gradient direction; different starting points but same end points and gradient direction; or same starting points, end points and gradient direction (Table 5). Among the criteria with different starting points, end points, and gradient direction the criteria-*Fscores* of individual wards differed and either increased, stayed the same or deteriorated with time. This shows that the CPOE implementation can result in different effects depending on the ward and its underlying process flows (Fig. 3).

## Discussion

This study showed that the implementation of a CPOE system—after adjusting for the influence of additional covariates—led to a substantial improvement of medication documentation quality. The novelty of these study results lies in their depth of detail that allows to draw direct conclusions with respect to the measures needed to further improve medication documentation quality.

Interestingly, two criteria deteriorated after introduction of electronic prescribing, namely the documentation of allergies and intolerances and the prescription of the active ingredients with abbreviations. Whereas a lot is known on the acceptance of allergy alerts [28], knowledge on changes in allergy documentation due to CPOE implementation is scarce; in two studies, CPOE implementation improved the documentation of allergies [29, 30]. The reason for this apparent discrepancy between these studies and our findings is unclear, which is why it is important to consider the underlying processes of allergy documentation. The deterioration in our setting may be due to the change in workflow from the hand-written documentation of allergies on paper to the structured entry in the CPOE system. On paper, allergies are entered as free text, whereas in the CPOE system, a structured entry of drugs or drug classes from an allergy list is needed. Further training measures for prescribers may improve acceptance of the allergy documentation tool contained in the CPOE system.

The second criterion showing a significant deterioration in the criteria-*Fscore* was criterion #9 (The prescription does not contain any abbreviations for the active substance). The use of abbreviations in drug prescribing is common—especially in handwritten prescriptions—and can easily lead to misinterpretation and even serious medication errors [31]. For this reason, there are a number of institutions, such as the Institute for Safe Medication Practices, that have published lists of error-prone abbreviations that should not be used in the communication of medical information [32]. The implemented CPOE system displays the prescribed drugs by their brand name. Surprisingly and unfortunately, manufacturers tend to use abbreviations in the trade names of their drugs, especially if they are generics, which explains the deterioration of the fulfilment value of criterion #9. Since it is difficult to influence the naming of drugs by manufacturers, one possible solution to the problem would be to always add the active ingredient to the display of prescriptions in the CPOE system.

The clinical relevance of the assessed criteria certainly varies; whether well-known abbreviations of active substances are a potential error source (e.g., 5-FU for fluorouracil or MCP for metoclopramide) is debatable, as is the absence of age in years (criterion #2) when the date of birth is clearly documented instead. However, we did not do any weighting according to the clinical relevance of the criteria due to the lack of validated standards. Our assessment was conservative in the sense that the predefined evaluation scheme was strictly followed and the calculation of *fulfilment scores* was based solely on the fulfilment and applicability of the criteria.

The study showed that different wards had variable prescription-*Fscores* often diverging from the global average of all wards. Additionally, the change patterns of individual criteria differed substantially between individual wards. This is most likely due to the different workflows of the respective wards, which had different general procedures at baseline. These procedures were harmonized through the introduction of the CPOE system. As an example, allergies or drug history taking differed between the pilot wards; it was either the nurse’s, the assistant’s, or the physician’s responsibility to enter allergies or drug history into the patient chart, whereas after CPOE implementation this task fell uniformly to the physician. Additionally, not only the templates for paper-based charts varied between the wards, this was also true for established documentation methods and comprehensibility, both of which were reflected in the degree of criteria fulfilment. Task switching and alteration of process flows due to CPOE implementation is common and the impact of CPOE on clinical workflow is known to be double-edged [33, 34]. It has been shown that users of CPOE systems may adopt work-arounds that are error-prone, if the system’s usability is poor or the handling is deemed cumbersome [35]. It is therefore important to closely monitor process changes, suggest improvements to clinical workflows, and assist clinical staff in adapting to the changes introduced by CPOE implementation. Furthermore, the continuous observation and follow-up on workflow changes is important in order to detect whether suggested adaptions resulted in an improvement or a deterioration.

The study has several limitations: First, despite aiming for comparable patient cohorts before and after implementation, the post-implementation cohort showed a smaller number of “total”, “as needed”, and “other” prescriptions. However, the multivariable analysis accounted for such imbalances suggesting that imbalances in prescription categories can be deemed negligible. Moreover, we only assessed the medication regimens of 320 patients at one time frame before and after implementation, which might limit the transferability of the results to other settings or other CPOE systems and neglects potential learning curves. We only adjusted for the influence of a number of well-known covariates. However, there might be other influential factors like the physician experience, physician workload, or physician attitude towards the CPOE system that may influence the quality of prescription documentation. Additionally, certain patient characteristics as age, sex, type of medication, clinical condition, diagnoses, or the time period of admission could affect the quality of prescription documentation. The hierarchical model with a random intercept on patient characteristics and adjustment for further variables accounts for such confounding influences whenever possible, although residual confounding cannot be ruled out. Another potential confounder may be distinct underlying prescribing workflows that may differ not only between wards, but even, on a smaller level, between different prescribers. Therefore, a precise analysis of workflows before and after CPOE implementation is needed, especially when there is a need to compensate for the negative effects of CPOE implementation. However, given the large magnitude of the CPOE effect estimate, the results can be considered as robust even with further potential confounders unavailable for adjustment. This is in line with a very large E-value of 19.7 corresponding to our effect estimate; this means that a (set of) unmeasured confounder(s) would have to increase the likelihood of improvement nearly 20-fold and would have to be as unequally distributed between the intervention and control group [36]. Ideally, the observed improvement in prescription documentation quality would also be translatable into improved patient outcomes. Whether this is the case in this setting should be subject of further prospective studies.

## Conclusions

This study provides a clear description of the influences of a CPOE system on detailed aspects of prescription documentation. It shows that the quality of prescription documentation increases substantially with the implementation of the CPOE system. However, there are also aspects—even in the documentation of the prescription—that are difficult to fulfil with a CPOE system. As the effects of a CPOE implementation have been proven double-edged, precise insights into the nature of effects is needed in order to derive improvement recommendations for CPOE systems and their usage. The in-depth analysis of distinct quality criteria allowed to identify specific issues where prescriber training, improvement of software or adaptation of clinical workflows can lead to a better use of the CPOE system and, potentially, to a further improvement of clinical documentation.

## Supplementary Information

**Additional file 1. **Assessment scheme for categorisation of prescriptions. Assessment scheme in accordance to the recommendation “Good prescribing practice in drug therapy” (APS). APS: alliance for patient safety; CPOE: computerized physician order entry; eGFR: estimated glomerular filtration rate.

## Data Availability

The datasets used and analysed during the current study are available from the corresponding author on reasonable request.

## Abbreviations

Change pattern with different starting points, end points, and gradient direction
Change pattern with different starting and end points but same gradient direction
Change pattern with different starting points but same end points and gradient direction
Change pattern with same starting points, end points, and gradient direction
APS: Alliance for patient safety
CDSS: Clinical decision support system
CI: Confidence interval
CPOE: Computerized physician order entry
Criteria-*Fscore*: Fulfilment score per criterion
EHR: Electronic health record
GLMM: Generalized linear mixed model
N: Number
OR: Odds ratio
P: *P * value
Prescription-*Fscore*: Fulfilment score per prescription
R: Pearson r correlation
SD: Standard deviation
